# Impact of an infectious diseases specialist-led antimicrobial stewardship programmes on antibiotic use and antimicrobial resistance in a large Korean hospital

**DOI:** 10.1038/s41598-018-33201-8

**Published:** 2018-10-03

**Authors:** Hyeonjun Hwang, Bongyoung Kim

**Affiliations:** 1 School of Economic Sciences, Washington State University, Pullman, USA; 2 Department of Internal Medicine, Hanyang University College of Medicine, Seoul, Korea

## Abstract

The aim of this study was to evaluate the impact of an infectious diseases specialist (IDS)-led antimicrobial stewardship programmes (ASPs) in a large Korean hospital. An interrupted time series analysis assessing the trends in antibiotic use and antimicrobial resistance rate of major pathogens between September 2015 and August 2017 was performed in an 859-bed university-affiliated hospital in Korea. The restrictive measure for designated antibiotics led by an IDS reduced carbapenems usage by −4.57 days of therapy (DOT)/1,000 patient-days per month in general wards (GWs) (95% confidence interval [CI], −6.69 to −2.46; *P* < 0.001), and by −41.50 DOT/1,000 patient-days per month in intensive care units (ICUs) (95% CI, −57.91 to −25.10; *P* < 0.001). Similarly, glycopeptides usage decreased by −2.61 DOT/1,000 patient-days per month in GWs (95% CI, −4.43 to −0.79; *P* = 0.007), and −27.41 DOT/1,000 patient-days per month in ICUs (95% CI, −47.03 to −7.79; *P* = 0.009). Use of 3^rd^ generation cephalosporins, beta-lactam/beta-lactamase inhibitors, and fluoroquinolones in GWs showed change comparable with that of carbapenems or glycopeptides use. Furthermore, trends of antimicrobial resistance rate of *Staphylococcus aureus* to gentamicin in GWs, *Staphylococcus aureus* to ciprofloxacin and oxacillin in ICUs, and *Pseudomonas aeruginosa* to imipenem in ICUs decreased in slope in the intervention period. The in-hospital mortality rate per 1,000 patient-days among ICU patients remained stable between the pre-intervention and intervention periods. In conclusion, an IDS-led ASPs could enact a meaningful reduction in antibiotic use, and a decrease in antibiotic resistance rate, without changing mortality rates in a large Korean hospital.

## Introduction

Antimicrobial resistance is one of the greatest threats to public health and is an emerging crisis for humans^1^. Once a pathogen acquires resistance to antibiotics, it renders antimicrobial treatment ineffective, leading to increased mortality, prolonged hospitalization, and increased medical costs^2^. In spite of this, in the last decade, few pharmaceutical companies have actively retained antibiotic discovery programmes^3^. To curb the spreading of antimicrobial resistance, the World Health Organization (WHO) established a global action plan to tackle antimicrobial resistance, which was endorsed by the Group of 7 (G7) summit in 2015^4^. Accordingly, the Korean Ministry of Health and Welfare established the Korean National Action Plan on Antimicrobial Resistance in 2016^5^.

Antimicrobial stewardship programmes (ASPs) are often a key strategy among action plans against antimicrobial resistance^4,5^. ASPs comprise a set of multidisciplinary activities focusing on proper antibiotic use, including implementing interventions for antibiotic prescription, monitoring of antibiotic usage and resistance patterns, regular reporting information on antibiotic use and resistance to medical staff, and educating clinicians about resistance and optimal prescription^6^.

While many large hospitals in Korea has been conducting ASPs since 2000s, they have been limited, and heavily dependent on modified preauthorization-of-antibiotic use programmes; in other words, restrictive measures for designated antibiotics^7^. The main reason for this has been the lack of manpower in the ASPs in most hospitals: ASPs were operated mainly by one or two infectious diseases specialists (IDSs) in each hospital^7^. Therefore, in order to improve ASPs in Korea, it is necessary to reinforce the priority of, and increase manpower to the implementation of ASPs.

The aim of this study was to evaluate the impact of an IDS-led ASPs in a large Korean hospital. To this end, we conducted an interrupted time series analysis on the antibiotic use and the antimicrobial resistance rate of major pathogens before, and after interventions.

## Material and Methods

### Study site

Eulji University hospital is an 859-bed university-affiliated secondary care hospital with a 34-bed intensive care unit (ICU). In addition, there is a 20-bed trauma unit and a 30-bed neonatal ICU; but no burn, or bone marrow transplant units.

There have been no actual ASPs implemented in the hospital except for occasional educational initiatives. There were no written internal guidelines regarding empirical antibiotic administration in common infectious diseases. Even though a computerized antibiotic control programme was developed in October 2005, it was not operational due to a lack of manpower: all requested antibiotics were approved without any restriction. Furthermore, frequent absence of the IDSs discouraged physicians from consulting on proper antibiotic use: there were no available IDSs on site during December 2010–March 2011, August 2011–September 2012, June 2014–July 2014, and January 2016–August 2016.

Infection control measures remained largely unchanged until September 2016. The most prominent strategy for infection control was monitoring and feedback of hand hygiene; average annual compliance rate was 70.8% in 2014, 68.6% in 2015, and 74.6% in 2016.

In September 2016, an IDS who had 1.5 years’ experience in running ASPs started his service at the hospital, and dedicated 2.5–3.5 hours per day to running the ASP.

### Major intervention: restrictive measure for designated antibiotics

In September 2016, restrictive measures for designated antibiotics were initiated, using a computerized antibiotic control programme. Designated antibiotics included carbapenems (imipenem, meropenem, ertapenem, and doripenem), tigecycline, glycopeptides (vancomycin and teicoplanin), oxazolidinone (linezolid), and polymyxin (colistin). Physicians were instructed to fill out a special antibiotic order form when prescription of any of the above antibiotics was needed. The purpose for the antibiotic use was a mandatory field on the special antibiotic order form. Once the special antibiotic order was made, the IDS could assess the prescription through the computerized antibiotic control programme. The decision on whether to approve or reject the prescription of the designated antibiotics was made via the programme within 48 hours, after medical record review. Pending the decision of IDS, the designated antibiotics could be administered to avoid delay in initiating therapy. The appropriateness of antibiotic use was assessed in accordance with the antibiograms of isolated pathogens, and the Sanford guideline to antimicrobial therapy^8^. For the approved antibiotics, follow-up evaluation for the appropriateness was performed after the period of approval which was set by the IDS: usually 4–7 days. Regardless of antibiotic order outcome, a written suggestion of appropriate antibiotics was sent to the prescribing physicians.

### Minor intervention – monitoring for unnecessary double anaerobic coverage prescription

In April 2017, a system for monitoring unnecessary double anaerobic coverage prescription was established. When combination antibiotic prescription with either metronidazole or clindamycin, and one of beta-lactam/beta-lactamase inhibitors (BL/BLIs) or carbapenems was detected via the computerized system, a pharmacist reviewed the medical records and sent the assessment of the appropriateness of each prescription to the IDS. The IDS confirmed the appropriateness as mentioned in a previous report by Song *et al*.^9^. If the combination prescription of anaerobic antibiotics was assessed as “inappropriate”, the pharmacist notified each attending physician about the result.

### Study design and data collection

The trends in antibiotic use, antimicrobial resistance rate for major pathogens, and in-hospital mortality before, and after interventions were analysed.

The primary outcome of the study was antibiotic use. The secondary outcomes consisted of the antimicrobial resistance rate for major pathogens, and in-hospital mortality. Patients in the general wards (GWs) and ICU were analysed separately, and the trauma unit, neonatal ICU, and paediatric ward were excluded from analysis. In-hospital mortality was analysed only for ICU patients.

Data on the number of monthly antibiotic prescriptions, monthly patient-days, and monthly antimicrobial sensitivity tests of major bacterial pathogens among inpatients between September 2015 and August 2017 were acquired from the electronic database. In addition, monthly Acute Physiology and Chronic Health Evaluation (APACHE 2) score, and monthly in-hospital mortality rate among ICU patients between September 2015 and August 2017 were acquired from the hospital data processing department.

The study protocol was approved by the Institutional Review Boards of the Eulji University Hospital (2017-12-001-001), and the requirement for written informed consent from patients was waived due to the retrospective nature of the study, and its impracticability.

### Antibiotics

In this paper, Anatomical Therapeutic Chemical (ATC) classification system class J01 antibiotics, which does not include antifungal agents or anti-tuberculosis agents, were included for analysis^10^. Systemic agents with per oral or parenteral administration routes were included, while topical agents were excluded. Each class of antibiotic was quantified via days of therapy (DOT), which was then standardized per 1,000 patient-days (PD)^11^.

We classified antibiotic agents into 19 classes: 1^st^ generation cephalosporins (1^st^ CEPs), 2^nd^ generation cephalosporins (2^nd^ CEPs), 3^rd^ generation cephalosporins (3^rd^ CEPs), 4^th^ generation cephalosporins (4^th^ CEPs), aminoglycosides (AGs), BL/BLIs, carbapenems, fluoroquinolones (FQs), glycopeptides, lincosamides, macrolides, metronidazole, monobactam, oxazolidinone, penicillins, polymyxin, tetracyclines, tigecycline, and trimethoprim/sulfamethoxazole (SXT). Other antibiotics, such as amphenicol, fosfomycin, and streptogramin were excluded because they are rarely used.

We defined 3^rd^ CEPs, 4^th^ CEPs, BL/BLIs, and FQs as broad-spectrum antibiotics. Carbapenems, tigecycline, glycopeptides, oxazolidinone, and polymyxin were defined as antibiotics against multidrug-resistant (MDR) pathogens. The remaining antibiotic classes were defined as non-broad-spectrum antibiotics.

### Antimicrobial resistance rate for major pathogens

We analysed the antimicrobial resistance rate for major bacterial pathogens isolated from any site of patients at least 48 hours after hospital admission. The first isolate of these pathogens for each admission per patient was included in the analysis. In addition, we performed subgroup analysis according to the type of specimens (blood, urine, and sputum).

In this paper, the major bacterial pathogens were: *Escherichia coli, Klebsiella pneumoniae, Acinetobacter baumanii, Pseudomonas aeruginosa, Staphylococcus aureus*, and *Enterococcus faecium*. We defined the antimicrobial resistance rate as the proportion of resistant isolates among total isolates.

All isolates and their antimicrobial susceptibilities were identified using a Vitek 2 automated bacterial identification system (bioMèrieux, Marcy-I’Etoile, France). The breakpoints of each compound were defined in reference to the Clinical and Laboratory Standards Institute (CLSI)^12^, and outcomes of R (resistance) or I (intermediate) were defined as resistance. Extended-spectrum beta-lactamase (ESBL)-producing isolates were defined as *E. coli* or *K. pneumoniae* proven to be positive by an ESBL test in the Vitek 2 system.

### Statistical analysis

The impact of intervention on antibiotic use, antimicrobial resistance rate, and in-hospital mortality were evaluated through segmented regression analysis of an interrupted time series with adjustment for autocorrelation^13^. We confirmed that Durbin-Watson test statistics for the overall antibiotic usage and resistance rate indicated no serious autocorrelation after the adjustment. The study period (September 2015–August 2017) was divided by interventions and analysed as follows:

(i) In the analysis of the impact by major intervention, the study period was divided into pre-intervention (September 2015–August 2016) and major intervention (September 2016–August 2017).
(ii) In the analysis of the impact by minor intervention, the study period was divided into pre-intervention (September 2015–August 2016), major intervention (September 2016–March 2017), and minor intervention (April 2017–August 2017).

The impact of minor intervention was tested against the whole previous period (September 2015–March 2017) because main target antibiotics of the minor intervention (lincosamides and metronidazole) were not included in designated antibiotics of the restrictive measures.

We defined “change in level” as the difference between the observed value at the beginning of the pre-intervention, and intervention periods, and “change in trend” as the difference between the change rates of the pre-intervention, and intervention periods. The segmented regression analysis was applied using the newey command (considering Newey-West standard errors) in STATA version 15 (StataCorp LLC., College Station, TX). Statistical significance was defined as *P* < 0.05.

## Results

### Impact of the major intervention on antibiotic use

Table 1 shows changing trends of antibiotic use after the major intervention. The total antibiotic use in GWs during the pre-intervention, and intervention periods were 1065.98, and 1103.71 DOT/1,000 PD, respectively. Although an immediate increase in use by 106.81 DOT/1,000 PD (*P* = 0.003) was observed after the intervention, the intervention resulted in a change in trend by −28.14 DOT/1,000 PD per month (*P* < 0.001). The total antibiotic use in ICUs were 3945.29, and 3313.13 DOT/1,000 PD in the pre-intervention, and intervention period, respectively; the secular trend did not change after the intervention.

**Table 1:** Changing trends of antibiotic use after the major intervention (restrictive measure for designated antibiotics).

	Change in level^a^	SE	95% CI	*P*	Change in trend^b^	SE	95% CI	*P*
**General wards**								
**Antibiotics against MDR pathogens**								
Carbapenems	**−39.11**	**8.70**	**(−57.25 to −20.97)**	<**0.001**	**−4.57**	**1.01**	**(−6.69 to −2.46)**	<**0.001**
Glycopeptides	**−20.23**	**7.53**	**(−35.93 to −4.53)**	**0.014**	**−2.61**	**0.87**	**(−4.43 to −0.79)**	**0.007**
Oxazolidinone	**−**0.49	1.43	(**−**3.47 to 2.50)	0.738	**−**0.23	0.17	(**−**0.59 to 0.12)	0.178
Polymyxin	**−3.69**	**1.57**	**(−6.96 to −0.42)**	**0.029**	**−**0.17	0.24	(**−**0.66 to 0.33)	0.493
Tigecycline	3.41	2.46	(**−**1.72 to 8.53)	0.181	0.22	0.27	(**−**0.35 to 0.80)	0.424
**Subtotal**	**−60.10**	**15.71**	**(−92.88 to −27.33)**	**0.001**	**−7.36**	**1.85**	**(−11.22 to −3.51)**	**0.001**
**Broad-spectrum antibiotics**								
3^rd^ CEPs	3.12	9.47	(**−**16.64 to 22.88)	0.745	**−2.66**	**1.18**	**(−5.13 to −0.19)**	**0.036**
4^th^ CEPs	**15.58**	**6.06**	**(2.95 to 28.22)**	**0.018**	**−**0.52	0.75	(**−**2.08 to 1.05)	0.500
BL/BLIs	**60.67**	**14.91**	**(29.57 to 91.77)**	**0.001**	**−5.63**	**2.70**	**(−11.27 to 0.02)**	**0.051**
FQs	**−**5.53	9.23	(**−**24.79 to 13.73)	0.556	**−5.59**	**1.78**	**(−9.31 to −1.88)**	**0.005**
**Subtotal**	**73.84**	**18.85**	**(34.52 to 113.16)**	**0.001**	**−14.34**	**2.74**	**(−20.12 to −8.67)**	<**0.001**
**Non-broad-spectrum antibiotics**								
1^st^ CEPs	**−**3.55	11.68	(**−**27.91 to 20.81)	0.764	**−**0.48	1.60	(**−**3.82 to 2.87)	0.770
2^nd^ CEPs	10.98	11.19	(**−**12.36 to 34.33)	0.338	1.53	1.26	(**−**1.10 to 4.16)	0.240
AGs	7.06	3.72	(**−**0.71 to 14.82)	0.073	**−**0.26	0.53	(**−**1.37 to 0.84)	0.627
Lincosamide	**7.92**	**2.83**	**(2.01 to 13.84)**	**0.011**	**−**0.592	0.31	(**−**1.25 to 0.06)	0.074
Macrolides	**46.62**	**17.34**	**(10.45 to 82.79)**	**0.014**	**−**1.13	2.51	(**−**6.37 to 4.12)	0.659
Metronidazole	4.11	10.93	(**−**18.70 to 26.92)	0.711	**−4.51**	**1.47**	**(−7.57 to −1.45)**	**0.006**
Monobactam	1.07	0.64	(**−**0.26 to 2.41)	0.109	**−0.16**	**0.08**	**(−0.33 to 0.00)**	**0.054**
Penicillins	4.68	2.45	(**−**0.43 to 9.80)	0.071	**−**0.09	0.29	(**−**0.71 to 0.52)	0.756
Tetracyclines	3.54	2.02	(**−**4.13 to 25.38)	0.094	**−**0.04	0.27	(**−**2.20 to 0.89)	0.892
SXT	10.62	7.07	(**−**0.67 to 7.75)	0.149	**−**0.65	0.74	(**−**0.60 to 0.53)	0.388
**Subtotal**	**93.07**	**29.95**	**(30.60 to 155.55)**	**0.006**	**−**6.38	3.87	(**−**14.46 to 1.70)	0.115
**Total**	**106.81**	**31.98**	**(40.10 to 173.51)**	**0.003**	**−28.14**	**4.49**	**(−37.51 to −18.78)**	<**0.001**
**Intensive care units**								
**Antibiotics against MDR pathogens**								
Carbapenems	**−484.92**	**62.82**	**(−651.97 to −353.87)**	<**0.001**	**−41.50**	**7.86**	**(−57.91 to −25.10)**	<**0.001**
Glycopeptides	**−331.62**	**71.40**	**(−480.56 to −182.69)**	<**0.001**	**−27.41**	**9.41**	**(−47.03 to −7.79)**	**0.009**
Oxazolidinone	**−**1.72	4.01	(**−**10.09 to 6.64)	0.672	0.96	0.79	(**−**0.69 to 2.62)	0.239
Polymyxin	**−52.12**	**22.71**	**(−99.50 to −4.74)**	**0.033**	**−7.53**	**3.02**	**(−13.83 to −1.24)**	**0.021**
Tigecycline	31.09	16.03	(**−**2.34 to 64.53)	0.067	2.83	2.17	(**−**1.71 to 7.37)	0.208
**Subtotal**	**−839.29**	**126.99**	**(−1104.19 to −574.40)**	<**0.001**	**−72.65**	**15.62**	**(−105.25 to −40.06)**	<**0.001**
**Broad-spectrum antibiotics**								
3^rd^ CEPs	**−**31.94	57.23	(**−**151.32 to 87.44)	0.583	7.96	8.14	(**−**9.03 to 24.95)	0.340
4^th^ CEPs	44.30	27.10	(**−**12.23 to 100.83)	0.118	1.19	3.43	(**−**5.96 to 8.34)	0.732
BL/BLIs	61.25	77.61	(**−**100.63 to 223.13)	0.439	**−**13.69	8.63	(**−**31.69 to 4.30)	0.128
FQs	**−350.25**	**92.91**	**(−544.06 to −156.44)**	**0.001**	5.79	12.52	(**−**20.31 to 31.90)	0.648
**Subtotal**	**−276.64**	**105.18**	**(−496.04 to −57.25)**	**0.016**	1.25	14.36	(**−**28.70 to 31.21)	0.931
**Non-broad-spectrum antibiotics**								
1^st^ CEPs	31.93	24.26	(**−**18.68 to 82.53)	0.203	**−**0.64	3.15	(**−**7.21 to 5.93)	0.842
2^nd^ CEPs	70.23	38.22	(**−**9.50 to 149.96)	0.081	**24.86**	**6.99**	**(10.29 to 39.44)**	**0.002**
AGs	**62.15**	**15.52**	**(29.77 to 94.52)**	**0.001**	2.16	2.90	(**−**3.90 to 8.21)	0.466
Lincosamide	**60.81**	**24.49**	**(9.74 to 111.89)**	**0.022**	**−**2.99	2.73	(**−**8.68 to 2.71)	0.287
Macrolides	**−**34.64	25.58	(**−**88.00 to 18.73)	0.191	**−**5.06	2.93	(**−**11.18 to 1.06)	0.100
Metronidazole	**−154.27**	**57.67**	**(−274.57 to −33.97)**	**0.015**	3.21	7.47	(**−**12.37 to 18.80)	0.672
Monobactam	**−**7.18	5.41	(**−**18.47 to 4.11)	0.200	**−**0.14	0.92	(**−**2.06 to 1.78)	0.879
Penicillins	**23.37**	**10.31**	**(1.86 to 44.88)**	**0.035**	**−**1.05	1.15	(**−**3.46 to 1.36)	0.374
Tetracyclines	**12.26**	**5.61**	**(3.94 to 34.57)**	**0.041**	0.03	0.64	(**−**2.62 to 4.65)	0.966
SXT	**19.26**	**7.34**	**(0.56 to 23.95)**	**0.016**	1.01	1.74	(**−**1.31 to 1.36)	0.567
**Subtotal**	83.91	71.19	(**−**64.60 to 232.42)	0.252	21.40	11.47	(**−**2.54 to 45.34)	0.077
**Total**	**−1032.02**	**213.29**	**(−1476.93 to −587.11)**	<**0.001**	**−**50.00	28.34	(**−**109.11 to 9.11)	0.093

^a^The unit for change in level is days of therapy (DOT)/1,000 patient-days; ^b^The unit for change in trend is DOT/1,000 patient-days per month.

Abbreviations: SE, Standard errors; CI, Confidence interval; MDR, multidrug-resistant; 3^rd^ CEPs, 3^rd^ generation cephalosporins; 4^th^ CEPs, 4^th^ generation cephalosporins; BL/BLIs, beta-lactam/beta-lactamase inhibitors; FQs, fluoroquinolones; 1^st^ CEPs, 1^st^ generation cephalosporins; 2^nd^ CEPs, 2^nd^ generation cephalosporins; AGs, aminoglycosides; SXT, trimethoprim/sulfamethoxazole.

**Figure 1:**
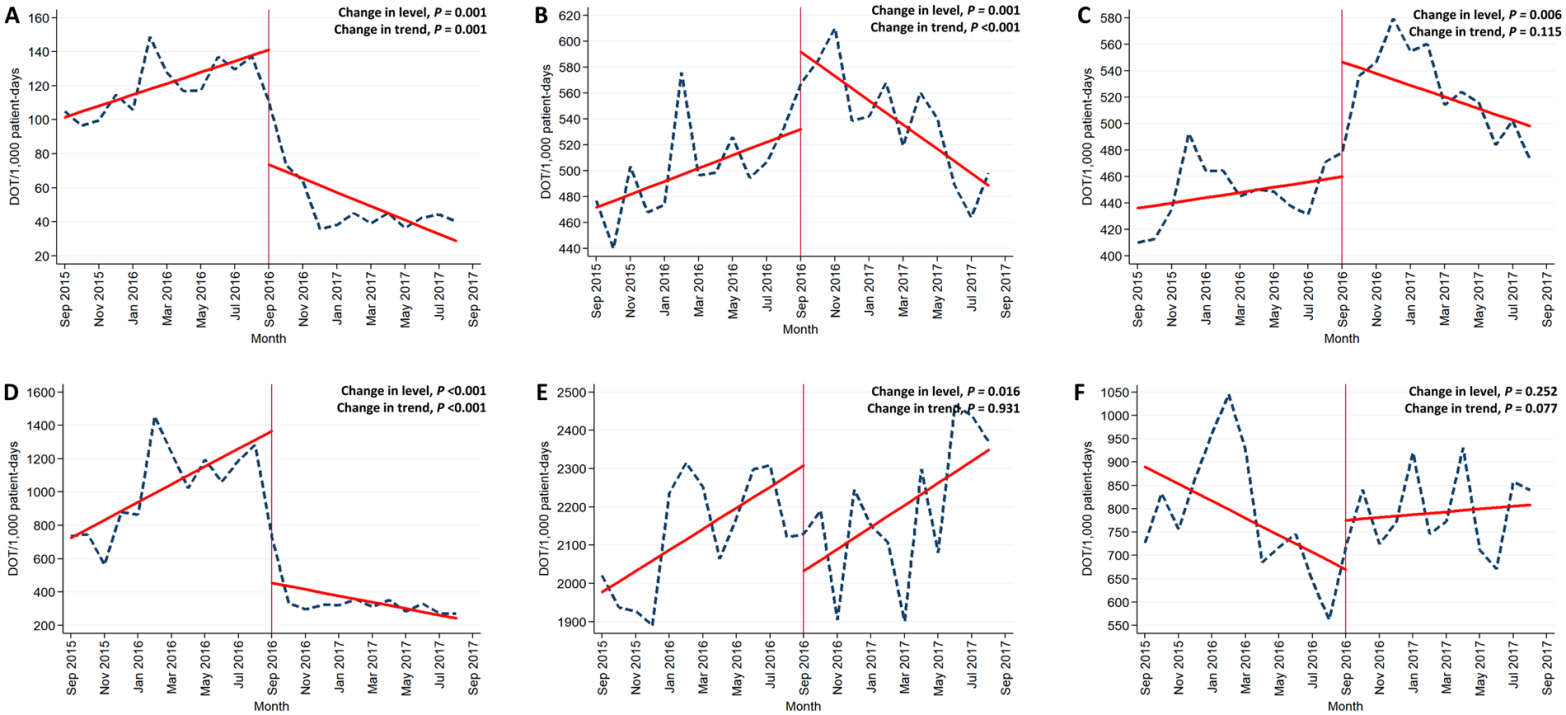
Changing trends in antibiotic use among inpatients over time. (**A**) Antibiotics against multidrug-resistant pathogens in general wards; (**B**) Broad-spectrum antibiotics in general wards; (**C**) Non-broad-spectrum antibiotics in general wards; (**D**) Antibiotics against multidrug-resistant pathogens in intensive care units; (**E**) Broad-spectrum antibiotics in intensive care units; (**F**) Non-broad-spectrum antibiotics in intensive care units.

Antibiotic usage against MDR pathogens was significantly affected by the intervention for patients in both the GWs and ICUs. The intervention resulted in a significant immediate decrease in use (−60.10 and −839.29 DOT/1,000 PD in GWs and ICUs, respectively) and a significant negative change in slope (−7.36, and −72.65 DOT/1,000 PD per month in GWs and ICUs, respectively). As for broad-spectrum antibiotics in GWs, there was a significant increase in level of antibiotic usage after the intervention (73.84 DOT/1,000 PD, *P* = 0.001), while a significant decrease in trend by −14.34 DOT/1,000 PD per month was observed (*P* < 0.001). For the usage of broad-spectrum antibiotics in ICUs, there was a significant decrease in level (−276.64 DOT/1,000 PD, *P* = 0.016), but no significant change in trend. The intervention did not affect the trend of non-broad-spectrum antibiotics for patients in both the GWs and ICUs (Fig. 1).

Among antibiotics against MDR pathogens, the impact of the major intervention on carbapenems and glycopeptides were particularly prominent. The restrictive measure for designated antibiotics immediately decreased the carbapenems usage by −39.11 DOT/1,000 PD in GWs (*P* < 0.001) and by −484.92 DOT/1,000 PD in ICUs (*P* < 0.001); the trend changed by −4.57 DOT/1,000 PD per month in GWs (*P* < 0.001) and by −41.50 DOT/1,000 PD per month in ICUs (*P* < 0.001). Similarly, glycopeptides usage was significantly affected by the intervention both in the GWs and ICUs: change in level were −20.23 DOT/1,000 PD in GWs (*P* = 0.014) and −331.62 DOT/1,000 PD in ICUs (*P* < 0.001); change in trend were −2.61 DOT/1,000 PD per month in GWs (*P* = 0.007) and −27.41 DOT/1,000 PD per month in ICUs (*P* = 0.009). Furthermore, the major intervention resulted in a significant decrease in both level and trend for the use of polymyxin among patients of ICUs.

As for broad-spectrum antibiotics, the impact of the intervention was more significant in GWs compared to ICUs. The intervention significantly decreased trends in the usage of 3^rd^ CEPs, BL/BLIs, and FQs in GWs. The usage of FQs in ICUs was significantly reduced immediately after the intervention, but no significant change in trend was observed.

The impact of restrictive measures for designated antibiotics on non-broad-spectrum antibiotics was not as significant as that on antibiotics against MDR pathogens, or broad-spectrum antibiotics. There was a significant immediate increase in the antibiotic usage of lincosamides and macrolides in GWs; AGs, lincosamide, penicillins, tetracyclines, and SXT in ICUs. However, there was no significant change in trend for most antibiotic classes except monobactam in GWs and 2^nd^ CEPs in ICUs.

### Impact of the minor intervention on antibiotic use

Table 2 shows changing trends of antibiotic use after the minor intervention. The monitoring for unnecessary double anaerobic coverage prescription changed the trend in metronidazole usage in GWs by −13.86 DOT/1,000 PD per month (*P* < 0.001) but not affected that in ICUs. Accordingly, there was a significant negative change in slope for the consumption of non-broad-spectrum antibiotics in GWs after the minor intervention (−17.40 DOT/1,000 PD per month, *P* = 0.038). The usage of carbapenems, BL/BLIs, and lincosamides were not affected by the minor intervention in both the GWs and ICUs.

**Table 2:** Changing trends of antibiotic use after the major intervention (restrictive measure for designated antibiotics) and the minor intervention (monitoring for unnecessary double anaerobic coverage prescription).

	Major intervention								Minor intervention			
	Change in level^a^	SE	95% CI	*P*	Change in trend^b^	SE	95% CI	*P*	Change in trend^b^	SE	95% CI	*P*
**General wards**												
**Antibiotics against MDR pathogens**												
Carbapenems	**−30.58**	**11.14**	**(−53.98 to −7.18)**	**0.013**	**−7.04**	**2.20**	**(−11.66 to −2.42)**	**0.005**	3.76	2.21	(**−**0.89 to 8.41)	0.106
Glycopeptides	**−**8.52	6.17	(**−**21.48 to 4.44)	0.184	**−6.01**	**1.24**	**(−8.62 to −3.40)**	<**0.001**	**4.99**	**1.39**	**(2.07 to 7.91)**	**0.002**
Oxazolidinone	0.15	1.98	(**−**4.02 to 4.31)	0.942	**−**0.43	0.36	(**−**1.19 to 0.33)	0.248	0.07	0.36	(**−**0.68 to 0.83)	0.841
Polymyxin	**−**3.48	2.05	(**−**7.78 to 0.82)	0.106	**−**0.14	0.37	(**−**0.92 to 0.63)	0.702	**1.19**	**0.55**	**(0.04 to 2.34)**	**0.043**
Tigecycline	4.96	3.82	(**−**3.06 to 12.98)	0.210	**−**0.24	0.65	(**−**1.62 to 1.13)	0.712	0.41	0.77	(**−**1.21 to 2.02)	0.601
**Subtotal**	**−37.47**	**15.69**	**(−70.44 to −4.50)**	**0.028**	**−13.87**	**3.20**	**(−20.59 to −7.16)**	<**0.001**	**10.43**	**3.31**	**(3.47 to 17.38)**	**0.006**
**Broad-spectrum antibiotics**												
3^rd^ CEPs	13.86	12.18	(**−**11.72 to 39.44)	0.270	**−6.09**	**2.32**	**(−10.95 to −1.22)**	**0.017**	0.52	2.47	(**−**4.67 to 5.72)	0.835
4^th^ CEPs	3.66	4.82	(**−**6.46 to 13.79)	0.457	**2.88**	**1.10**	**(0.55 to 5.20)**	**0.018**	**−5.95**	**1.22**	**(−8.51 to −3.38)**	<**0.001**
BL/BLIs	**50.30**	**16.2**	**(16.26 to 84.33)**	**0.006**	**−**2.83	3.39	(**−**9.95 to 4.28)	0.414	**−**7.23	3.96	(**−**15.55 to 1.10)	0.085
FQs	1.54	8.57	(**−**16.46 to 19.55)	0.859	**−7.77**	**2.18**	**(−12.35 to −3.20)**	**0.002**	1.40	3.67	(**−**6.32 to 9.11)	0.708
**Subtotal**	**69.37**	**23.35**	**(20.30 to 118.44)**	**0.008**	**−13.82**	**4.57**	**(−23.41 to −4.22)**	**0.007**	**−**11.25	8.66	(**−**29.45 to 6.94)	0.210
**Non-broad-spectrum antibiotics**												
1^st^ CEPs	**−**0.84	15.79	(**−**34.02 to 32.34)	0.958	**−**0.61	2.89	(**−**6.68 to 5.46)	0.836	**9.65**	**3.52**	**(2.26 to 17.04)**	**0.013**
2^nd^ CEPs	**−**1.76	13.63	(**−**30.39 to 26.88)	0.899	5.12	2.70	(**−**0.55 to 10.80)	0.074	**−4.80**	**2.74**	**(−12.56 to −1.03)**	**0.023**
AGs	3.19	3.72	(**−**4.63 to 11.02)	0.402	0.64	0.65	(**−**0.73 to 2.00)	0.341	**−4.54**	**1.58**	**(−7.86 to −1.22)**	**0.01**
Lincosamide	5.72	3.38	(**−**1.38 to 12.82)	0.108	0.09	0.79	(**−**1.56 to 1.75)	0.906	**−**0.33	0.95	(**−**2.33 to 1.66)	0.728
Macrolides	38.26	21.62	(**−**7.17 to 83.69)	0.084	1.30	4.51	(**−**8.19 to 10.78)	0.777	**−**3.58	4.08	(**−**12.15 to 4.98)	0.391
Metronidazole	**−**9.34	14.54	(**−**39.90 to 21.22)	0.529	**−**1.23	2.32	(**−**6.12 to 3.65)	0.603	**−13.86**	**2.61**	**(−19.34 to −8.38)**	<**0.001**
Monobactam	1.68	0.89	(**−**0.19 to 3.55)	0.076	**−0.34**	**0.15**	**(−0.65 to −0.02)**	**0.036**	0.26	0.17	(**−**0.10 to 0.62)	0.146
Penicillins	4.16	3.81	(**−**3.84 to 12.17)	0.289	0.14	0.70	(**−**1.34 to 1.62)	0.845	0.83	0.88	(**−**1.01 to 2.67)	0.357
Tetracyclines	4.01	3.25	(**−**9.36 to 33.62)	0.233	**−**0.13	0.65	(**−**4.56 to 2.31)	0.847	0.80	0.66	(**−**3.52 to 3.86)	0.238
SXT	12.13	10.23	(**−**2.82 to 10.85)	0.251	**−**1.13	1.63	(**−**1.50 to 1.24)	0.499	0.17	1.76	(**−**0.58 to 2.19)	0.925
**Subtotal**	57.22	34.63	(**−**15.54 to 129.99)	0.116	3.86	7.84	(**−**12.62 to 20.34)	0.629	**−17.40**	**7.78**	**(−33.74 to −1.06)**	**0.038**
**Total**	**89.12**	**42.46**	**(−0.09 to 178.34)**	**0.050**	**−23.83**	**9.40**	**(−43.59 to −4.08)**	**0.021**	**−**18.23	10.86	(**−**41.05 to 4.59)	0.111
**Intensive care units**												
**Antibiotics against MDR pathogens**												
Carbapenems	**−449.90**	**91.91**	**(−642.99 to −256.81)**	<**0.001**	**−51.89**	**18.53**	**(−90.83 to −12.95)**	**0.012**	12.08	18.56	(**−**26.90 to 51.07)	0.523
Glycopeptides	**−299.73**	**90.48**	**(−489.83 to−109.64)**	**0.004**	**−37.64**	**16.06**	**(−71.37 to −3.90)**	**0.031**	0.94	14.55	(**−**29.63 to 31.51)	0.949
Oxazolidinone	**−**3.33	3.97	(**−**11.68 to 5.02)	0.413	0.92	1.16	(**−**1.52 to 3.35)	0.439	**−7.35**	**1.42**	**(−10.33 to −4.38)**	<**0.001**
Polymyxin	**−**53.60	34.19	(**−**125.43 to 18.24)	0.134	**−**6.57	6.04	(**−**19.27 to 6.13)	0.291	6.32	6.98	(**−**8.34 to 20.98)	0.377
Tigecycline	49.93	25.86	(**−**10.41 to 98.26)	0.107	**−**0.38	5.14	(**−**11.18 to 10.42)	0.942	12.18	6.12	(**−**0.67 to 25.04)	0.062
**Subtotal**	**−762.64**	**178.52**	**(−1137.70 to −387.58)**	<**0.001**	**−95.56**	**32.5**	**(−163.83 to −27.29)**	**0.009**	24.18	31.59	(**−**42.18 to 90.54)	0.454
**Broad-spectrum antibiotics**												
3^rd^ CEPs	59.89	82.56	(**−**113.57 to 233.35)	0.478	**−**18.95	17.00	(**−**54.68 to 16.77)	0.280	35.8	20.14	(**−**6.51 to 78.11)	0.092
4^th^ CEPs	2.89	22.77	(**−**44.96 to 50.74)	0.900	13.82	7.49	(**−**1.92 to 29.56)	0.082	**−**9.74	8.90	(**−**28.44 to 8.95)	0.288
BL/BLIs	49.61	108.13	(**−**177.35 to 276.57)	0.652	**−**8.96	17.04	(**−**44.75 to 26.84)	0.606	12.71	17.54	(**−**24.15 to 49.57)	0.478
FQs	**−216.75**	**85.87**	**(−397.15 to −36.35)**	**0.021**	**−34.8**	**13.95**	**(−64.12 to −5.49)**	**0.023**	32.93	25.43	(**−**20.50 to 86.36)	0.212
**Subtotal**	**−**104.36	112.45	(**−**340.62 to 131.90)	0.366	**−48.89**	**21.78**	**(−94.66 to −3.13)**	**0.038**	71.69	37.45	(**−**6.98 to 150.37)	0.072
**Non-broad-spectrum antibiotics**												
1^st^ CEPs	**64.69**	**23.56**	**(15.20 to 114.18)**	**0.013**	**−9.47**	**4.10**	**(−18.09 to −0.86)**	**0.033**	**22.74**	**5.35**	**(11.50 to 33.98)**	<**0.001**
2^nd^ CEPs	47.10	51.63	(**−**61.36 to 155.56)	0.374	**31.26**	**11.35**	**(7.43 to 55.10)**	**0.013**	**−**13.91	20.24	(**−**56.44 to 28.62)	0.501
AGs	**40.68**	**15.16**	**(8.82 to 72.54)**	**0.015**	**6.18**	**2.72**	**(0.47 to 11.89)**	**0.035**	**−37.87**	**8.53**	**(−55.79 to −19.94)**	<**0.001**
Lincosamide	22.77	21.56	(**−**22.51 to 68.06)	0.305	8.69	4.83	(**−**1.47 to 18.84)	0.089	**−**7.99	5.05	(**−**18.60 to 2.62)	0.131
Macrolides	**−**17.82	34.18	(**−**89.64 to 53.99)	0.608	**−**9.47	6.27	(**−**22.64 to 3.70)	0.148	13.34	7.99	(**−**3.45 to 30.12)	0.112
Metronidazole	**−**119.90	73.71	(**−**274.77 to 34.96)	0.121	**−**6.88	14.69	(**−**37.74 to 23.98)	0.645	13.14	21.9	(**−**32.87 to 59.16)	0.556
Monobactam	**−**7.11	5.69	(**−**19.06 to 4.83)	0.227	0.00	1.15	(**−**2.42 to 2.42)	1.000	2.23	1.51	(**−**1.06 to 5.30)	0.179
Penicillins	10.34	8.48	(**−**7.47 to 28.15)	0.238	2.66	2.81	(**−**3.26 to 8.57)	0.357	**−**6.52	4.55	(**−**16.08 to 3.04)	0.169
Tetracyclines	3.53	7.10	(8.36 to 47.87)	0.625	2.63	2.24	(**−**7.02 to 4.60)	0.256	**−**2.85	2.57	(**−**3.87 to 20.45)	0.283
SXT	**28.11**	**9.40**	**(−11.39 to 18.45)**	**0.008**	**−**1.21	2.76	(**−**2.08 to 7.33)	0.667	8.29	5.79	(**−**8.25 to 2.56)	0.169
**Subtotal**	72.39	79.28	(**−**94.17 to 238.95)	0.373	24.39	15.36	(**−**7.88 to 56.66)	0.130	**−**9.51	34.50	(**−**81.99 to 62.97)	0.786
**Total**	**−794.61**	**223.29**	**(−1263.73 to −325.49)**	**0.002**	**−120.06**	**36.17**	**(−196.06 to −44.06)**	**0.004**	86.36	68.56	(**−**57.68 to 230.39)	0.224

^a^The unit for change in level is days of therapy (DOT)/1,000 patient-days; ^b^The unit for change in trend is DOT/1,000 patient-days per month.

Abbreviations: SE, Standard errors; CI, Confidence interval; MDR, multidrug-resistant; 3^rd^ CEPs, 3^rd^ generation cephalosporins; 4^th^ CEPs, 4^th^ generation cephalosporins; BL/BLIs, beta-lactam/beta-lactamase inhibitors; FQs, fluoroquinolones; 1^st^ CEPs, 1^st^ generation cephalosporins; 2^nd^ CEPs, 2^nd^ generation cephalosporins; AGs, aminoglycosides; SXT, trimethoprim/sulfamethoxazole.

### Impact of the major intervention on the antimicrobial resistance rate in major bacterial pathogens

**Figure 2:**
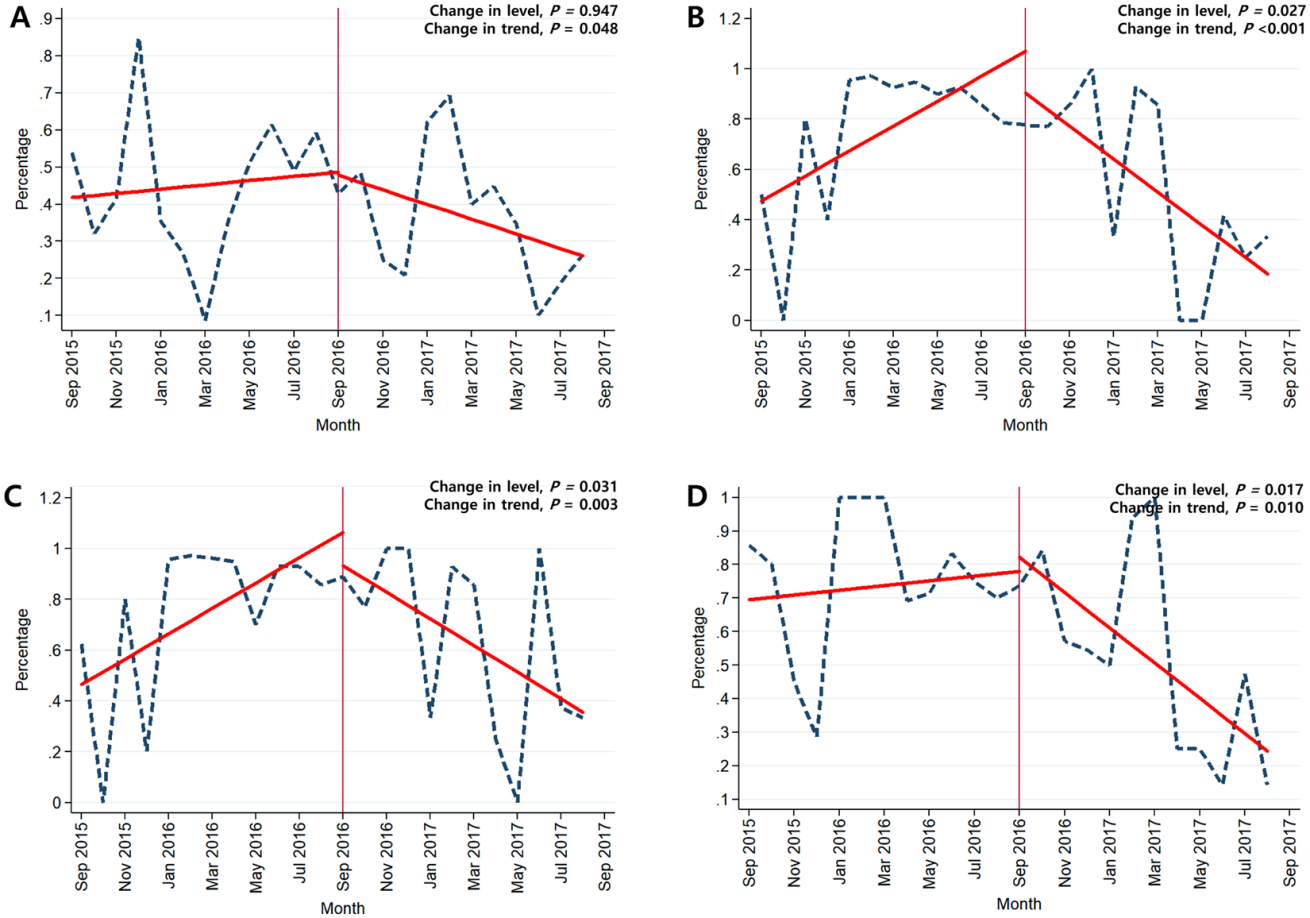
Changing trends in antimicrobial resistance over time. (**A**) Resistant rate of *Staphylococcus aureus* to gentamicin in general wards; (**B**) Resistant rate of *Staphylococcus aureus* to ciprofloxacin in intensive care units; (**C**) Resistant rate of *Staphylococcus aureus* to oxacillin in intensive care units; (**D**) Resistance rate of *Pseudomonas aeruginosa* to imipenem in intensive care units.

Table 3 shows a changing pattern in antimicrobial resistance rate in major bacterial pathogens after the major intervention. The intervention effect was prominent for the resistance rate of *S. aureus* isolates: a decrease in trend was observed for gentamicin in GWs (−0.028% per month, *P* = 0.048), ciprofloxacin in ICUs (−0.115% per month, *P* < 0.001), and oxacillin in ICUs (−0.012% per month, *P* = 0.003). In addition, a significant decrease in trend was observed for the resistance rate of *P. aeruginosa* to imipenem in ICUs (−0.049% per month, *P* = 0.010) (Fig. 2).

**Table 3:** Changing trend of resistance rate to the indicated agent in major bacterial pathogens after the major intervention (restrictive measure for designated antibiotics).

	Pre-intervention period	Intervention period	Change in level^a^	SE	95% CI	*P*	Change in trend^b^	SE	95% CI	*P*
**General wards**										
***Escherichia coli***										
Ciprofloxacin (%)	304/473 (64.3)	364/551 (66.1)	0.083	0.095	(−0.116 to 0.282)	0.394	−0.025	0.014	(−0.054 to 0.003)	0.078
Gentamicin (%)	170/473 (35.9)	258/551 (46.8)	0.071	0.102	(−0.142 to 0.284)	0.494	−0.013	0.015	(−0.045 to 0.019)	0.419
ESBL production (%)	203/472 (43.0)	291/550 (52.9)	0.124	0.088	(−0.060 to 0.308)	0.176	−0.017	0.012	(−0.041 to 0.007)	0.164
Imipenem (%)	1/473 (0.2)	1/551 (0.2)	0.001	0.008	(−0.016 to 0.018)	0.902	−0.001	0.001	(−0.003 to 0.001)	0.187
***Klebsiella pneumoniae***										
Ciprofloxacin (%)	98/194 (50.5)	131/261 (50.2)	−0.037	0.127	(−0.302 to 0.228)	0.775	−0.004	0.023	(−0.052 to 0.043)	0.851
Gentamicin (%)	38/194 (19.6)	35/261 (13.4)	−0.091	0.094	(−0.287 to 0.104)	0.341	−0.009	0.015	(−0.040 to 0.022)	0.538
ESBL production (%)	102/194 (52.6)	127/261 (48.7)	0.173	0.136	(−0.111 to 0.457)	0.219	−0.022	0.018	(−0.059 to 0.015)	0.221
Imipenem (%)	0/194 (0.0)	9/261 (3.4)								
***Acinetobacter baumanii***										
Ciprofloxacin (%)	183/206 (88.8)	154/177 (87.0)	0.073	0.117	(−0.170 to 0.315)	0.540	0.004	0.020	(−0.038 to 0.045)	0.860
Gentamicin (%)	175/206 (85.0)	110/177 (62.1)	−0.225	0.140	(−0.515 to 0.065)	0.122	0.033	0.020	(−0.009 to 0.075)	0.119
Cefepime (%)	193/206 (93.7)	160/177 (90.4)	0.048	0.056	(−0.069 to 0.164)	0.407	0.006	0.011	(−0.018 to 0.029)	0.630
Imipenem (%)	181/206 (83.1)	147/177 (83.1)	−0.009	0.117	(−0.253 to 0.235)	0.939	0.011	0.020	(−0.031 to 0.054)	0.588
***Pseudomonas aeruginosa***										
Ciprofloxacin (%)	202/306 (66.0)	89/271 (32.8)	−**0.166**	**0.070**	**(**−**0.311 to** −**0.020)**	**0.028**	−0.023	0.015	(−0.054 to 0.008)	0.143
Gentamicin (%)	185/306 (60.5)	61/271 (22.5)	−**0.204**	**0.093**	**(**−**0.399 to** −**0.010)**	**0.040**	−0.016	0.018	(−0.053 to 0.021)	0.377
Cefepime (%)	303/303 (100)	270/270 (100)	—	—	—	—	—	—	—	—
Imipenem (%)v	218/306 (71.2)	78/271 (28.8)	−**0.208**	**0.093**	**(**−**0.402 to** −**0.014)**	**0.037**	−0.026	0.016	(−0.059 to 0.006)	0.104
***Staphylococcus aureus***										
Ciprofloxacin (%)	437/519 (84.2)	160/178 (89.9)	−**0.246**	**0.114**	**(**−**0.483 to** −**0.009)**	**0.043**	−0.017	0.017	(−0.052 to 0.018)	0.327
Gentamicin (%)	224/519 (43.2)	166/178 (93.3)	0.008	0.113	(−0.227 to 0.243)	0.947	−**0.028**	**0.013**	**(**−**0.056 to 0.000)**	**0.048**
Oxacillin (%)	458/517 (88.6)	62/178 (34.8)	−0.146	0.122	(−0.399 to 0.107)	0.242	−0.006	0.015	(−0.036 to 0.025)	0.698
***Enterococcus faecium***										
Ampicillin (%)	135/147 (91.8)	160/178 (89.9)	0.021	0.270	(−0.541 to 0.584)	0.938	0.041	0.034	(−0.029 to 0.112)	0.232
Ciprofloxacin (%)	139/147 (94.6)	166/178 (93.3)	−0.013	0.169	(−0.365 to 0.339)	0.938	0.010	0.023	(−0.039 to 0.058)	0.680
Vancomycin (%)	83/147 (56.5)	62/178 (34.8)	−0.083	0.200	(−0.498 to 0.332)	0.681	0.011	0.034	(−0.059 to 0.082)	0.739
**Intensive care units**										
***Escherichia coli***										
Ciprofloxacin (%)	28/55 (50.9)	61/79 (77.2)	0.273	0.282	(−0.316 to 0.862)	0.346	0.019	0.037	(−0.058 to 0.097)	0.606
Gentamicin (%)	15/55 (27.3)	47/79 (59.5)	0.205	0.317	(−0.456 to 0.866)	0.524	0.045	0.042	(−0.044 to 0.133)	0.303
ESBL production (%)	28/55 (50.9)	55/78 (70.5)	0.218	0.277	(−0.360 to 0.795)	0.441	0.055	0.039	(−0.027 to 0.136)	0.176
Imipenem (%)	0/55 (0.0)	0/79 (0.0)	—	—	—	—	—	—	—	—
***Klebsiella pneumoniae***										
Ciprofloxacin (%)	34/68 (50.0)	49/86 (57.0)	0.125	0.260	(−0.416 to 0.667)	0.634	−0.029	0.032	(−0.097 to 0.038)	0.371
Gentamicin (%)	4/68 (5.9)	0/86 (0.0)	−0.036	0.040	(−0.119 to 0.047)	0.377	0.002	0.006	(−0.010 to 0.014)	0.706
ESBL production (%)	34/68 (50.0)	52/86 (60.5)	0.169	0.280	(−0.414 to 0.753)	0.551	−0.026	0.034	(−0.097 to 0.046)	0.461
Imipenem (%)	1/68 (1.5)	0/86 (0.0)	−0.026	0.029	(−0.087 to 0.035)	0.391	−0.001	0.002	(−0.005 to 0.004)	0.686
***Acinetobacter baumanii***										
Ciprofloxacin (%)	335/341 (98.2)	229/235 (97.4)	0.004	0.042	(−0.083 to 0.092)	0.918	0.006	0.005	(−0.005 to 0.017)	0.291
Gentamicin (%)	323/341 (94.7)	184/235 (78.3)	−**0.313**	**0.149**	**(**−**0.624 to** −**0.001)**	**0.049**	0.028	0.018	(−0.010 to 0.066)	0.141
Cefepime (%)	336/341 (98.5)	229/235 (97.4)	−0.031	0.028	(−0.089 to 0.027)	0.274	−0.001	0.004	(−0.010 to 0.008)	0.817
Imipenem (%)	332/341 (97.4)	226/235 (96.2)	0.004	0.046	(−0.092 to 0.100)	0.926	0.004	0.006	(−0.010 to 0.017)	0.582
***Pseudomonas aeruginosa***										
Ciprofloxacin (%)	63/108 (58.3)	116/164 (70.7)	0.186	0.139	(−0.105 to 0.476)	0.198	0.002	0.020	(−0.041 to 0.044)	0.936
Gentamicin (%)	59/108 (54.6)	92/164 (56.1)	0.142	0.139	(−0.147 to 0.432)	0.317	−0.031	0.024	(−0.081 to 0.018)	0.204
Cefepime (%)	108/108 (100)	164/164 (100)			—				—	
Imipenem (%)v	75/108 (69.4)	98/164 (59.8)	0.137	0.105	(−0.082 to 0.356)	0.208	−**0.049**	**0.017**	**(**−**0.085 to** −**0.013)**	**0.010**
***Staphylococcus aureus***										
Ciprofloxacin (%)	155/177 (87.6)	56/88 (63.6)	−0.052	0.154	(−0.372 to 0.269)	0.741	−**0.115**	**0.027**	**(**−**0.171 to** −**0.059)**	**0.000**
Gentamicin (%)	95/177 (53.7)	35/88 (39.8)	0.020	0.255	(−0.512 to 0.552)	0.938	0.001	0.043	(−0.090 to 0.092)	0.981
Oxacillin (%)	156/177 (88.1)	67/88 (76.1)	−0.029	0.148	(−0.338 to 0.280)	0.845	−**0.102**	**0.031**	**(**−**0.166 to** −**0.039)**	**0.003**
***Enterococcus faecium***										
Ampicillin (%)	25/28 (89.3)	23/23 (100)	0.249	0.266	(−0.325 to 0.823)	0.366	0.017	0.032	(−0.052 to 0.085)	0.605
Ciprofloxacin (%)	25/28 (89.3)	23/23 (100)	0.249	0.266	(−0.325 to 0.823)	0.366	0.017	0.032	(−0.052 to 0.085)	0.605
Vancomycin (%)	15/28 (53.6)	11/23 (47.8)	0.095	0.361	(−0.685 to 0.876)	0.796	0.049	0.063	(−0.086 to 0.185)	0.445

^a^The unit for change in level is antimicrobial resistance rate (%); ^b^The unit for change in trend is antimicrobial resistance rate (%) per month.

Abbreviations: SE, Standard errors; CI, Confidence interval; ESBL, Extended-spectrum beta-lactamase.

According to the subgroup analysis, there was a significant negative change in slope for ciprofloxacin resistance rate of *E. coli*, isolated from sputum in both the GWs and ICUs (−0.036%, and −0.111% per month in GWs and ICUs, respectively). Furthermore, as for *K. pneumoniae* isolated from sputum in ICUs, a significant decrease in trend was observed for the rate of ciprofloxacin resistance (−0.273% per month, *P* = 0.033) and ESBL production (−0.356% per month, *P* = 0.016) (Supplementary Tables 2 and 3).

### Impact of the major intervention on in-hospital mortality among ICU patients

The average APACHE 2 scores of the pre-intervention, and intervention period were 17.5, and 20.8 per patient, respectively; there was no significant change in the level (coefficient −0.537, *P* = 0.766) and trend (coefficient 0.404, *P* = 0.171) of APACHE 2 score after the intervention.

The average in-hospital mortality rates per 1,000 patient-days were 19.4 in the pre-intervention period, and 18.6 in the intervention period. In-hospital mortality among ICU patients remained stable between the two periods: there was no significant change in level (coefficient 0.007, *P* = 0.862) and trend (coefficient 0.004, *P* = 0.476).

## Discussion

These results show that a significant reduction in antibiotic use, and a decrease in antimicrobial resistance rates were achieved by an IDS-driven ASP in a large hospital in Korea. IDSs are a key qualified resource to develop and lead ASPs across all healthcare settings^14^. The effect of IDS-driven ASPs is well established by several studies: a significant improvement in the appropriateness of antibiotic prescription, and a decreased antibiotic consumption were commonly found^15,16^. A recent study in the UK found that antibiotic therapy was 30% lower in the IDS-led group compared with other medical teams, with no adverse clinical outcome^17^. In addition to the effect on antibiotic use, the IDS-driven ASPs decreased mortality, reduced length of hospitalization, and reduced the incidence of MDR pathogens^16^.

As with most large hospitals in Korea, the major intervention at this study site was restrictive measures for designated antibiotics^7^; which is similar to the preauthorization-of-antibiotic use programme in that the prescription of certain antibiotics is restricted unless approval is granted. The efficacy of restrictive antibiotic measures on both the reduction of antibiotic usage, and a decrease in the incidence of MDR pathogens is well established^18,19^.

However, there are several potential drawbacks to restrictive measures. Firstly, a large proportion of the total usage of systemic antibiotics cannot be controlled properly by restrictive measures. We found that designated antibiotics comprise 9.6% of total systemic antibiotics in the study hospital. The designated antibiotics in the study hospital are commonly controlled in large hospitals in Korea; carbapenems, tigecycline, glycopeptides, oxazolidione, and polymyxin^7^. Secondly, some authors demonstrated that it may delay in initiating therapy and may result in a breakdown in trust and communication between physicians^6,20^. Thirdly, the effect on the reduction of antibiotic use declines gradually with the passage of time^21^.

Interestingly, our study not only showed a significant reduction in the usage of designated antibiotics, but also a significant reduction in usage of other broad-spectrum antibiotics, such as 3^rd^ CEPs, BL/BLIs, and FQs. This may be attributable to the effect of the written suggestion for appropriate antibiotic usage, which was given with the outcome of the decision on the prescription of designated antibiotics. Similar to these findings, the result of a recent single-centre-based study in Italy showed a mixed educational and restrictive measure for designated antibiotics resulted in reduction of both designated and undesignated antibiotic use^22^. Furthermore, a previous single-centre-based study in Korea showed that even though the compliance rate of attending physicians was low after antibiotic advisory consultation, a significant reduction in antibiotic use was observed^23^. In fact, non-restrictive feedback measures such as prospective audit and feedback are considered to be another key strategy for intervention measures of ASPs^6^. A recent study in the US revealed that a non-restrictive feedback measure (post-prescription review with feedback) was superior to a restrictive measure such as preauthorization-of-antibiotic use^24^. Therefore, reinforcement of feedback measures should be considered for better ASPs in Korean hospitals.

Unfortunately, ASPs in Korean hospitals heavily depended on restrictive measures for designated antibiotics due to limited manpower^7^. ASPs were operated only by one or two IDSs in 85.2% of large hospitals in Korea without appropriate reward for performing ASPs^7^. According to Infectious Disease Society of America (IDSA) guidelines, multidisciplinary antimicrobial stewardship teams, which include an IDS and a clinical pharmacist with infectious diseases training are essential for realizing ASPs, and individuals of the team should be compensated appropriately for their time^6^. Because ASPs are the most important and effective strategy for controlling MDR pathogens^25^, stakeholders and policy makers should consider a national level of support to implement appropriate ASPs in Korean hospitals.

The strength of the present study is that the study setting well reflects the real-world situation in large Korean hospitals. Another strength is that antibiotic usage was measured by DOT. To the best of our knowledge, this is the first study in Korea to record antibiotic usage by DOT. According to a recent guideline for ASPs, using DOT is recommended to measure antibiotic consumption^6^.

Despite the above strengths, there are some limitations to the present study. Firstly, due to the nature of the ecological study, the effects of confounders such as other ASPs, infection control measures, behavioural changes of physicians, etc. could not be fully controlled. Secondly, the patient-level clinical outcomes to the interventions could not be evaluated. Thirdly, this study was conducted in a large university-affiliated secondary care hospital. Therefore, the results cannot be generalized to different settings. Finally, the study focused on one IDS. Although the training system of IDSs is similar across Korean hospitals, and the quality of IDSs is controlled by the Korean Association of Internal Medicine; replicating the findings with more IDSs is required before these findings can be generalized.

## Conclusion

An IDS-led ASPs could enact a meaningful reduction in antibiotic use, and a decrease in antibiotic resistance rate, without changing mortality rates in a large Korean hospital. Further researches are necessary to assess the impact of IDS-led ASPs, with different methods, in different settings.

## Electronic supplementary material

Supplementary tables
