# Supplementary material for: Impact of an infectious diseases specialist-led antimicrobial stewardship programmes on antibiotic use and antimicrobial resistance in a large Korean hospital
Source: Sci Rep. 2018 Oct 3;8:14757. doi: 10.1038/s41598-018-33201-8 (PMC6170479; doi:10.1038/s41598-018-33201-8)
Supplement: Supplementary file 1 — Supplementary tables [file 41598_2018_33201_MOESM1_ESM.docx]

**Original article**

**Impact of an infectious diseases specialist-led antimicrobial stewardship programmes on antibiotic use and antimicrobial resistance in a large Korean hospital**

Hyeonjun Hwang^1^, Bongyoung Kim^2*^

^1^School of Economic Sciences, Washington State University, Pullman, USA

^2^Department of Internal Medicine, Hanyang University College of Medicine, Seoul, Korea

Supplementary Table 1. Consumption of antimicrobial agents for systemic use among inpatients (unit: DOT/1,000 patient-days)

A) General wards

|  | Pre-intervention period | | |  | Intervention period | | |
| --- | --- | --- | --- | --- | --- | --- | --- |
|  | Sep-Dec, 2015 | Jan-Apr, 2016 | May-Aug, 2016 |  | Oct-Dec, 2016 | Jan-Apr, 2017 | May-Aug, 2017 |
| **Antibiotics against MDR pathogens** |  |  |  |  |  |  |  |
| Carbapenems | 51.72 | 57.08 | 68.95 |  | 19.18 | 12.56 | 8.18 |
| Glycopeptides | 47.64 | 57.19 | 54.50 |  | 30.01 | 22.02 | 24.12 |
| Oxazolidinone | 0.45 | 1.81 | 1.27 |  | 0.28 | 0.89 | 0.76 |
| Polymyxin | 3.24 | 6.00 | 5.07 |  | 1.19 | 2.15 | 1.93 |
| Tigecycline | 1.06 | 2.00 | 0.50 |  | 5.95 | 4.25 | 5.95 |
| **Subtotal** | 104.12 | 124.09 | 130.30 |  | 56.61 | 41.87 | 40.94 |
| **Broad-spectrum antibiotics** |  |  |  |  |  |  |  |
| 3^rd^ CEPs | 176.08 | 183.90 | 181.47 |  | 173.59 | 162.33 | 157.45 |
| 4^th^ CEPs | 6.27 | 8.59 | 9.65 |  | 25.52 | 32.46 | 20.92 |
| BL/BLIs | 123.87 | 136.58 | 128.35 |  | 190.00 | 175.82 | 152.10 |
| FQs | 165.37 | 180.28 | 195.73 |  | 183.02 | 176.19 | 167.51 |
| **Subtotal** | 471.59 | 509.35 | 515.21 |  | 572.14 | 546.81 | 497.98 |
| **Non-broad-spectrum antibiotics** |  |  |  |  |  |  |  |
| 1^st^ CEPs | 143.04 | 142.92 | 163.38 |  | 178.35 | 171.00 | 191.37 |
| 2^nd^ CEPs | 91.57 | 106.88 | 95.68 |  | 113.47 | 132.88 | 126.91 |
| AGs | 17.45 | 18.62 | 17.46 |  | 23.60 | 26.95 | 20.14 |
| Lincosamide | 4.34 | 4.54 | 3.68 |  | 11.61 | 7.62 | 4.72 |
| Macrolides | 106.37 | 85.50 | 59.41 |  | 93.44 | 59.51 | 33.88 |
| Metronidazole | 63.86 | 85.27 | 95.56 |  | 98.00 | 116.61 | 94.94 |
| Monobactam | 0.43 | 1.22 | 0.35 |  | 1.60 | 0.41 | 0.07 |
| Penicillins | 0.34 | 1.16 | 0.91 |  | 8.02 | 5.85 | 5.34 |
| Tetracyclines | 8.67 | 6.26 | 6.71 |  | 10.95 | 7.31 | 7.23 |
| SXT | 2.95 | 3.61 | 3.89 |  | 16.54 | 9.21 | 9.13 |
| **Subtotal** | 439.04 | 456.00 | 447.04 |  | 555.58 | 537.35 | 493.73 |
| **Total** | 1014.75 | 1089.45 | 1092.55 |  | 1184.34 | 1126.04 | 1032.66 |

B) Intensive care units

|  | Pre-intervention period | | |  | Intervention period | | |
| --- | --- | --- | --- | --- | --- | --- | --- |
|  | Sep-Dec, 2015 | Jan-Apr, 2016 | May-Aug, 2016 |  | Oct-Dec, 2016 | Jan-Apr, 2017 | May-Aug, 2017 |
| **Antibiotics against MDR pathogens** |  |  |  |  |  |  |  |
| Carbapenems | 376.03 | 535.54 | 637.44 |  | 114.55 | 109.43 | 72.26 |
| Glycopeptides | 292.13 | 521.14 | 455.94 |  | 139.36 | 162.23 | 148.23 |
| Oxazolidinone | 3.76 | 3.37 | 1.76 |  | 0 | 11.52 | 9.76 |
| Polymyxin | 27.26 | 69.50 | 81.08 |  | 15.99 | 32.64 | 16.26 |
| Tigecycline | 33.61 | 12.82 | 7.03 |  | 48.95 | 19.92 | 42.52 |
| **Subtotal** | 732.78 | 1142.37 | 1183.26 |  | 318.86 | 335.73 | 289.03 |
| **Broad-spectrum antibiotics** |  |  |  |  |  |  |  |
| 3^rd^ CEPs | 569.45 | 610.44 | 518.57 |  | 502.28 | 441.56 | 503.72 |
| 4^th^ CEPs | 25.38 | 39.14 | 39.33 |  | 90.73 | 136.31 | 114.08 |
| BL/BLIs | 468.15 | 609.09 | 679.19 |  | 845.30 | 848.81 | 899.39 |
| FQs | 881.55 | 959.06 | 987.03 |  | 683.42 | 688.98 | 817.61 |
| **Subtotal** | 1944.54 | 2217.72 | 2224.13 |  | 2121.74 | 2115.67 | 2334.80 |
| **Non-broad-spectrum antibiotics** |  |  |  |  |  |  |  |
| 1^st^ CEPs | 69.80 | 94.69 | 45.70 |  | 91.38 | 68.15 | 88.29 |
| 2^nd^ CEPs | 262.51 | 223.57 | 118.21 |  | 200.06 | 235.66 | 209.57 |
| AGs | 22.09 | 25.86 | 4.61 |  | 58.42 | 91.91 | 49.49 |
| Lincosamide | 12.92 | 3.15 | 4.83 |  | 60.70 | 48.48 | 9.06 |
| Macrolides | 92.13 | 142.15 | 112.50 |  | 83.22 | 47.28 | 59.71 |
| Metronidazole | 291.19 | 383.94 | 355.09 |  | 205.94 | 288.94 | 306.23 |
| Monobactam | 4.70 | 16.64 | 6.81 |  | 6.20 | 1.92 | 2.79 |
| Penicillins | 0.23 | 0.22 | 0 |  | 21.54 | 27.84 | 4.88 |
| Tetracyclines | 5.40 | 5.17 | 0 |  | 15.34 | 12.96 | 4.41 |
| SXT | 34.08 | 10.12 | 19.34 |  | 38.51 | 22.08 | 35.78 |
| **Subtotal** | 795.06 | 905.53 | 667.11 |  | 781.33 | 845.21 | 770.21 |
| **Total** | 3472.38 | 4265.63 | 4074.49 |  | 3221.93 | 3296.62 | 3394.05 |

Abbreviations: MDR, multidrug-resistant; 3^rd^ CEPs, 3^rd^ generation cephalosporins; 4^th^ CEPs, 4^th^ generation cephalosporins; BL/BLIs, beta-lactam/beta-lactamase inhibitors; FQs, fluoroquinolones; 1^st^ CEPs, 1^st^ generation cephalosporins; 2^nd^ CEPs, 2^nd^ generation cephalosporins; AGs, aminoglycosides; SXT, trimethoprim/sulfamethoxazole

Supplementary Table 2. Resistance rate to the indicated agent in major bacterial pathogens isolated from patients in general wards according to specimen types.

A) Blood isolates

|  | Pre-intervention period | Intervention period | Change in level | SE | 95% CI | *P* | Change  in trend | SE | 95% CI | *P* |
| --- | --- | --- | --- | --- | --- | --- | --- | --- | --- | --- |
| ***Escherichia coli*** |  |  |  |  |  |  |  |  |  |  |
| Ciprofloxacin (%) | 49/98 (50.0) | 68/111 (61.3) | 0.252 | 0.290 | (-0.356 to 0.861) | 0.395 | -0.020 | 0.035 | (-0.094 to 0.053) | 0.569 |
| Gentamicin (%) | 18/98 (18.4) | 58/111 (52.3) | 0.142 | 0.322 | (-0.534 to 0.818) | 0.665 | -0.042 | 0.042 | (-0.131 to 0.047) | 0.334 |
| ESBL production (%) | 31/98 (31.6) | 62/111 (55.9) | 0.405 | 0.300 | (-0.226 to 1.036) | 0.194 | -0.023 | 0.039 | (-0.105 to 0.058) | 0.553 |
| Imipenem (%) | 1/98 (1.0) | 1/111 (0.9) | - | - | - | - | - | - | - | - |
| ***Klebsiella pneumoniae*** |  |  |  |  |  |  |  |  |  |  |
| Ciprofloxacin (%) | 9/21 (42.9) | 14/26 (53.8) | -0.302 | 0.566 | (-1.640 to 1.037) | 0.611 | -0.021 | 0.092 | (-0.238 to 0.197) | 0.828 |
| Gentamicin (%) | 7/21 (33.3) | 1/26 (3.8) | 0.026 | 0.255 | (-0.578 to 0.629) | 0.923 | 0.072 | 0.069 | (-0.092 to 0.236) | 0.332 |
| ESBL production (%) | 10/21 (47.6) | 8/26 (30.8) | -0.177 | 0.561 | (-1.503 to 1.150) | 0.762 | 0.045 | 0.104 | (-0.200 to 0.291) | 0.676 |
| Imipenem (%) | 0/21 (0.0) | 0/26 (0.0) | - | - | - | - | - | - | - | - |
| ***Acinetobacter baumanii*** |  |  |  |  |  |  |  |  |  |  |
| Ciprofloxacin (%) | 7/12 (58.3) | 6/7 (85.7) | 0.777 | 0.535 | (-0.532 to 2.086) | 0.197 | -0.042 | 0.072 | (-0.218 to 0.135) | 0.585 |
| Gentamicin (%) | 7/12 (58.3) | 6/7 (85.7) | 0.777 | 0.535 | (-0.533 to 2.086) | 0.197 | -0.042 | 0.072 | (-0.218 to 0.135) | 0.585 |
| Cefepime (%) | 8/12 (66.7) | 7/7 (100) | 0.376 | 0.325 | (-0.419 to 1.171) | 0.291 | 0.027 | 0.047 | (-0.087 to 0.141) | 0.585 |
| Imipenem (%) | 7/12 (58.3) | 4/7 (57.1) | 0.480 | 0.516 | (-0.783 to 1.743) | 0.388 | -0.047 | 0.070 | (-0.218 to 0.124) | 0.527 |
| ***Pseudomonas aeruginosa*** |  |  |  |  |  |  |  |  |  |  |
| Ciprofloxacin (%) | 4/6 (66.7) | 1/8 (12.5) | -1.104 | 1.248 | (-16.965 to 14.757) | 0.539 | -0.085 | 0.128 | (-1.705 to 1.535) | 0.625 |
| Gentamicin (%) | 2/6 (33.3) | 0/8 (0.0) | -1.048 | 1.248 | (-16.910 to 14.813) | 0.555 | -0.113 | 0.128 | (-1.733 to 1.507) | 0.539 |
| Cefepime (%) | 6/6 (100) | 8/8 (100) | - | - | - | - | - | - | - | - |
| Imipenem (%)v | 2/6 (33.3) | 2/8 (25.0) | -1.159 | 1.248 | (-17.021 to 14.702) | 0.523 | -0.057 | 0.128 | (-1.678 to 1.563) | 0.731 |
| ***Staphylococcus aureus*** |  |  |  |  |  |  |  |  |  |  |
| Ciprofloxacin (%) | 94/121 (77.7) | 34/111 (30.6) | -0.529 | 0.267 | (-1.092 to 0.034) | 0.064 | 0.027 | 0.046 | (-0.069 to 0.123) | 0.562 |
| Gentamicin (%) | 49/121 (40.5) | 18/111 (16.2) | -0.414 | 0.285 | (-1.016 to 0.187) | 0.164 | **-0.089** | **0.039** | **(-0.172 to -0.006)** | **0.036** |
| Oxacillin (%) | 101/121 (83.5) | 75/111 (67.6) | -0.264 | 0.243 | (-0.776 to 0.248) | 0.292 | 0.002 | 0.035 | (-0.072 to 0.075) | 0.965 |
| ***Enterococcus faecium*** |  |  |  |  |  |  |  |  |  |  |
| Ampicillin (%) | 8/12 (66.7) | 39/51 (76.5) | 0.445 | 0.299 | (-0.232 to 1.122) | 0.171 | **0.196** | **0.041** | **(0.103 to 0.289)** | **0.001** |
| Ciprofloxacin (%) | 12/12 (100) | 43/51 (84.3) | - | - | - | - | - | - | - | - |
| Vancomycin (%) | 8/12 (66.7) | 6/51 (11.8) | 0.194 | 0.242 | (-0.354 to 0.743) | 0.443 | **0.147** | **0.032** | **(0.074 to 0.219)** | **0.001** |

B) Urine isolates

|  | Pre-intervention period | Intervention period | Change in level | SE | 95% CI | *P* | Change  in trend | SE | 95% CI | *P* |
| --- | --- | --- | --- | --- | --- | --- | --- | --- | --- | --- |
| ***Escherichia coli*** |  |  |  |  |  |  |  |  |  |  |
| Ciprofloxacin (%) | 16/239 (70.7) | 45/212 (68.4) | -0.023 | 0.088 | (-0.207 to 0.162) | 0.800 | **-0.036** | **0.016** | **(-0.069 to -0.002)** | **0.037** |
| Gentamicin (%) | 101/239 (42.3) | 94/212 (44.3) | 0.097 | 0.122 | (-0.157 to 0.350) | 0.436 | 0.008 | 0.024 | (-0.043 to 0.059) | 0.743 |
| ESBL production (%) | 110/238 (46.2) | 106/212 (50.0) | 0.085 | 0.149 | (-0.226 to 0.396) | 0.574 | -0.001 | 0.021 | (-0.045 to 0.043) | 0.966 |
| Imipenem (%) | 0/239 (0.0) | 0/212 (0.0) | - | - | - | - | - | - | - | - |
| ***Klebsiella pneumoniae*** |  |  |  |  |  |  |  |  |  |  |
| Ciprofloxacin (%) | 25/50 (50.0) | 29/60 (48.3) | 0.613 | 0.323 | (-0.062 to 1.288) | 0.073 | 0.074 | 0.041 | (-0.011 to 0.159) | 0.084 |
| Gentamicin (%) | 12/50 (24.0) | 7/60 (11.7) | 0.015 | 0.148 | (-0.294 to 0.325) | 0.919 | 0.025 | 0.021 | (-0.020 to 0.069) | 0.261 |
| ESBL production (%) | 27/50 (54.0) | 21/60 (35.0) | 0.081 | 0.344 | (-0.638 to 0.801) | 0.815 | 0.082 | 0.043 | (-0.009 to 0.172) | 0.074 |
| Imipenem (%) | 0/50 (0.0) | 1/60 (1.7) |  |  | - |  |  |  | - |  |
| ***Acinetobacter baumanii*** |  |  |  |  |  |  |  |  |  |  |
| Ciprofloxacin (%) | 21/29 (72.4) | 18/20 (90.0) | 0.382 | 0.305 | (-0.264 to 1.029) | 0.228 | -0.035 | 0.050 | (-0.142 to 0.072) | 0.499 |
| Gentamicin (%) | 21/29 (72.4) | 13/20 (65.0) | -0.694 | 0.336 | (-1.407 to 0.019) | 0.056 | 0.078 | 0.058 | (-0.045 to 0.200) | 0.197 |
| Cefepime (%) | 27/29 (93.1) | 18/20 (90.0) | 0.151 | 0.275 | (-0.431 to 0.734) | 0.589 | 0.032 | 0.033 | (-0.037 to 0.101) | 0.342 |
| Imipenem (%) | 21/29 (72.4) | 16/20 (80.0) | -0.036 | 0.430 | (-0.947 to 0.876) | 0.935 | 0.008 | 0.062 | (-0.124 to 0.140) | 0.904 |
| ***Pseudomonas aeruginosa*** |  |  |  |  |  |  |  |  |  |  |
| Ciprofloxacin (%) | 73/96 (76.0) | 23/52 (44.2) | -0.346 | 0.289 | (-0.948 to 0.255) | 0.244 | -0.018 | 0.039 | (-0.098 to 0.062) | 0.646 |
| Gentamicin (%) | 71/96 (74.0) | 25/52 (48.1) | -0.264 | 0.287 | (-0.862 to 0.333) | 0.368 | -0.007 | 0.036 | (-0.082 to 0.069) | 0.859 |
| Cefepime (%) | 96/96 (100) | 52/52 (100) |  |  | - |  |  |  | - |  |
| Imipenem (%)v | 73/96 (76.0) | 19/52 (36.5) | -0.368 | 0.262 | (-0.913 to 0.177) | 0.175 | -0.030 | 0.034 | (-0.101 to 0.040) | 0.383 |
| ***Staphylococcus aureus*** |  |  |  |  |  |  |  |  |  |  |
| Ciprofloxacin (%) | 46/52 (88.5) | 24/27 (88.9) | 0.043 | 0.162 | (-0.305 to 0.391) | 0.794 | -0.046 | 0.055 | (-0.163 to 0.072) | 0.420 |
| Gentamicin (%) | 12/52 (23.1) | 11/27 (40.7) | 0.185 | 0.335 | (-0.534 to 0.903) | 0.590 | **-0.115** | **0.049** | **(-0.221 to -0.010)** | **0.034** |
| Oxacillin (%) | 46/52 (88.5) | 25/27 (92.6) | -0.151 | 0.137 | (-0.445 to 0.142) | 0.288 | **-0.130** | **0.031** | **(-0.196 to 0.064)** | **0.001** |
| ***Enterococcus faecium*** |  |  |  |  |  |  |  |  |  |  |
| Ampicillin (%) | 51/51 (100) | 53/55 (96.4) | - | - | - | - | - | - | **-** | - |
| Ciprofloxacin (%) | 51/51 (100) | 53/55 (96.4) | - | - | - | - | - | - | - | - |
| Vancomycin (%) | 18/51 (35.3) | 22/55 (40.0) | 0.551 | 0.325 | (-0.129 to 1.232) | 0.106 | 0.060 | 0.047 | (-0.038 to 0.157) | 0.214 |

C) Sputum isolates

|  | Pre-intervention period | Intervention period | Change in level | SE | 95% CI | *P* | Change  in trend | SE | 95% CI | *P* |
| --- | --- | --- | --- | --- | --- | --- | --- | --- | --- | --- |
| ***Escherichia coli*** |  |  |  |  |  |  |  |  |  |  |
| Ciprofloxacin (%) | 25/28 (89.3) | 67/95 (70.5) | 0.005 | 0.093 | (-0.190 to 0.200) | 0.959 | -0.027 | 0.017 | (-0.063 to 0.009) | 0.136 |
| Gentamicin (%) | 8/28 (28.6) | 58/95 (61.1) | 0.041 | 0.127 | (-0.223 to 0.306) | 0.748 | 0.005 | 0.024 | (-0.044 to 0.054) | 0.835 |
| ESBL production (%) | 23/28 (82.1) | 62/94 (66.0) | 0.057 | 0.148 | (-0.253 to 0.366) | 0.706 | 0.001 | 0.023 | (-0.047 to 0.049) | 0.952 |
| Imipenem (%) | 0/28 (0.0) | 0/95 (0.0) | - | - | - | - | - | - | - | - |
| ***Klebsiella pneumoniae*** |  |  |  |  |  |  |  |  |  |  |
| Ciprofloxacin (%) | 48/71 (67.6) | 71/116 (61.2) | 0.323 | 0.277 | (-0.258 to 0.905) | 0.258 | **0.094** | **0.035** | **(0.020 to 0.167)** | **0.015** |
| Gentamicin (%) | 13/71 (18.3) | 21/116 (18.1) | -0.012 | 0.151 | (-0.330 to 0.306) | 0.939 | 0.026 | 0.022 | (-0.020 to 0.072) | 0.259 |
| ESBL production (%) | 48/71 (67.6) | 73/116 (62.9) | 0.263 | 0.328 | (-0.427 to 0.953) | 0.434 | 0.041 | 0.040 | (-0.044 to 0.126) | 0.321 |
| Imipenem (%) | 0/71 (0.0) | 1/116 (0.9) | 0.010 | 0.013 | (-0.017 to 0.037) | 0.454 | 0.000 | 0.001 | (-0.002 to 0.003) | 0.763 |
| ***Acinetobacter baumanii*** |  |  |  |  |  |  |  |  |  |  |
| Ciprofloxacin (%) | 80/86 (93.0) | 56/66 (84.8) | 0.067 | 0.361 | (-0.701 to 0.835) | 0.855 | 0.007 | 0.049 | (-0.098 to 0.112) | 0.887 |
| Gentamicin (%) | 78/86 (90.7) | 45/66 (68.2) | **-0.775** | **0.319** | **(-1.455 to -0.096)** | **0.028** | **0.107** | **0.044** | **(0.012 to 0.201)** | **0.029** |
| Cefepime (%) | 82/86 (95.3) | 58/66 (87.9) | 0.062 | 0.356 | (-0.696 to 0.820) | 0.865 | 0.037 | 0.044 | (-0.057 to 0.130) | 0.420 |
| Imipenem (%) | 80/86 (93.0) | 54/66 (81.8) | -0.377 | 0.432 | (-1.298 to 0.545) | 0.397 | 0.056 | 0.057 | (-0.065 to 0.177) | 0.338 |
| ***Pseudomonas aeruginosa*** |  |  |  |  |  |  |  |  |  |  |
| Ciprofloxacin (%) | 103/143 (72.0) | 43/129 (33.3) | -0.332 | 0.288 | (-0.931 to 0.266) | 0.261 | -0.014 | 0.038 | (-0.092 to 0.064) | 0.713 |
| Gentamicin (%) | 92/143 (64.3) | 25/129 (19.4) | -0.250 | 0.286 | (-0.844 to 0.344) | 0.392 | -0.003 | 0.035 | (-0.076 to 0.071) | 0.942 |
| Cefepime (%) | 140/140 (100) | 128/128 (100) | - | - | - | - | - | - | - | - |
| Imipenem (%)v | 107/143 (74.8) | 38/129 (29.5) | -0.358 | 0.261 | (-0.900 to 0.184) | 0.184 | -0.026 | 0.033 | (-0.095 to 0.042) | 0.432 |
| ***Staphylococcus aureus*** |  |  |  |  |  |  |  |  |  |  |
| Ciprofloxacin (%) | 163/170 (95.9) | 86/103 (83.5) | 0.223 | 0.276 | (-0.356 to 0.802) | 0.429 | -0.027 | 0.045 | (-0.122 to 0.068) | 0.559 |
| Gentamicin (%) | 77/170 (45.3) | 62/103 (60.2) | 0.504 | 0.333 | (-0.196 to 1.205) | 0.148 | -0.046 | 0.049 | (-0.149 to 0.056) | 0.357 |
| Oxacillin (%) | 164/170 (96.5) | 93/103 (90.3) | 0.043 | 0.164 | (-0.300 to 0.387) | 0.794 | -0.065 | 0.034 | (-0.137 to 0.007) | 0.076 |
| ***Enterococcus faecium*** |  |  |  |  |  |  |  |  |  |  |
| Ampicillin (%) | 12/12 (100) | 13/13 (100) | 0.175 | 0.126 | (-0.088 to 0.437) | 0.181 | 0.005 | 0.018 | (-0.032 to 0.042) | 0.788 |
| Ciprofloxacin (%) | 12/12 (100) | 13/13 (100) | -0.017 | 0.034 | (-0.088 to 0.055) | 0.633 | -0.014 | 0.012 | (-0.039 to 0.010) | 0.232 |
| Vancomycin (%) | 6/11 (50.0) | 8/13 (61.5) | 0.367 | 0.310 | (-0.278 to 1.013) | 0.250 | 0.050 | 0.047 | (-0.047 to 0.146) | 0.298 |

^a^ The unit for change in level is antimicrobial resistance rate (%); the unit for change in trend is antimicrobial resistance rate (%) per month

^b^ Abbreviations: SE, Standard errors; CI, Confidence interval; ESBL, Extended-spectrum beta-lactamase

Supplementary Table 3. Resistance rate to the indicated agent in major bacterial pathogens isolated from inpatients in intensive care units according to specimen types.

A) Blood isolates

|  | Pre-intervention period | Intervention period | Change in level | SE | 95% CI | *P* | Change  in trend | SE | 95% CI | *P* |
| --- | --- | --- | --- | --- | --- | --- | --- | --- | --- | --- |
| ***Escherichia coli*** |  |  |  |  |  |  |  |  |  |  |
| Ciprofloxacin (%) | 6/13 (46.2) | 5/16 (31.3) | -0.852 | 0.663 | (-2.694 to 0.989) | 0.268 | 0.013 | 0.098 | (-0.257 to 0.284) | 0.897 |
| Gentamicin (%) | 0/13 (0.0) | 5/16 (31.3) | - | - | - | - | - | - | - | - |
| ESBL production (%) | 6/13 (46.2) | 9/16 (56.3) | 0.099 | 0.577 | (-1.503 to 1.701) | 0.872 | 0.094 | 0.110 | (-0.212 to 0.400) | 0.442 |
| Imipenem (%) | 0/13 (0.0) | 0/16 (0.0) | - | - | - | - | - | - | - | - |
| ***Klebsiella pneumoniae*** |  |  |  |  |  |  |  |  |  |  |
| Ciprofloxacin (%) | 2/12 (16.7) | 0/11 (0.0) | -0.773 | 0.476 | (-2.095 to 0.549) | 0.180 | -0.117 | 0.077 | (-0.330 to 0.095) | 0.200 |
| Gentamicin (%) | 0/12 (0.0) | 0/11 (0.0) | - | - | - | - | - | - | - | - |
| ESBL production (%) | 2/12 (16.7) | 0/11 (0.0) | -0.462 | 0.706 | (-2.422 to 1.499) | 0.549 | -0.127 | 0.093 | (-0.385 to 0.131) | 0.245 |
| Imipenem (%) | 0/12 (0.0) | 0/11 (0.0) | - | - | - | - | - | - | - | - |
| ***Acinetobacter baumanii*** |  |  |  |  |  |  |  |  |  |  |
| Ciprofloxacin (%) | 32/35 (91.4) | 34/37 (91.9) | 0.166 | 0.345 | (-0.579 to 0.910) | 0.639 | 0.064 | 0.044 | (-0.032 to 0.160) | 0.174 |
| Gentamicin (%) | 31/35 (88.6) | 28/37 (75.7) | -0.056 | 0.381 | (-0.878 to 0.767) | 0.886 | 0.081 | 0.047 | (-0.020 to 0.181) | 0.106 |
| Cefepime (%) | 35/35 (100) | 34/37 (91.9) | -0.175 | 0.190 | (-0.584 to 0.235) | 0.374 | 0.016 | 0.018 | (-0.024 to 0.055) | 0.402 |
| Imipenem (%) | 32/35 (91.4) | 34/37 (91.9) | 0.166 | 0.345 | (-0.579 to 0.910) | 0.639 | 0.064 | 0.044 | (-0.032 to 0.160) | 0.174 |
| ***Pseudomonas aeruginosa*** |  |  |  |  |  |  |  |  |  |  |
| Ciprofloxacin (%) | 8/26 (30.8) | 7/7 (100) | **0.822** | **0.248** | **(0.236 to 1.409)** | **0.013** | 0.038 | 0.069 | (-0.125 to 0.202) | 0.596 |
| Gentamicin (%) | 8/26 (30.8) | 6/7 (85.7) | **1.048** | **0.285** | **(0.376 to 1.721)** | **0.008** | -0.044 | 0.080 | (-0.234 to 0.147) | 0.605 |
| Cefepime (%) | 26/26 (100) | 7/7 (100) | - | - | - | - | - | - | - | - |
| Imipenem (%)v | 17/26 (65.4) | 5/7 (71.4) | 0.606 | 0.285 | (-0.069 to 1.281) | 0.071 | -0.135 | 0.074 | (-0.311 to 0.042) | 0.114 |
| ***Staphylococcus aureus*** |  |  |  |  |  |  |  |  |  |  |
| Ciprofloxacin (%) | 16/19 (84.2) | 4/5 (80.0) | **-1.271** | **0.284** | **(-2.174 to -0.368)** | **0.021** | -0.048 | 0.062 | (-0.245 to 0.148) | 0.491 |
| Gentamicin (%) | 9/19 (47.4) | 0/5 (0.0) | -0.123 | 0.226 | (-0.842 to 0.596) | 0.623 | 0.004 | 0.035 | (-0.108 to 0.117) | 0.915 |
| Oxacillin (%) | 17/19 (89.5) | 5/5 (100.0) | -0.212 | 0.366 | (-1.377 to 0.953) | 0.603 | -0.167 | 0.080 | (-0.420 to 0.087) | 0.127 |
| ***Enterococcus faecium*** |  |  |  |  |  |  |  |  |  |  |
| Ampicillin (%) | 5/6 (83.3) | 9/9 (100) | - | - | - | - | - | - | - | - |
| Ciprofloxacin (%) | 5/6 (83.3) | 9/9 (100) | - | - | - | - | - | - | - | - |
| Vancomycin (%) | 2/6 (33.3) | 0/9 (0.0) | -0.969 | 0.480 | (-2.498 to 0.559) | 0.137 | -0.112 | 0.055 | (-0.288 to 0.063) | 0.134 |

B) Urine isolates

|  | Pre-intervention period | Intervention period | Change in level | SE | 95% CI | *P* | Change  in trend | SE | 95% CI | *P* |
| --- | --- | --- | --- | --- | --- | --- | --- | --- | --- | --- |
| ***Escherichia coli*** |  |  |  |  |  |  |  |  |  |  |
| Ciprofloxacin (%) | 9/18 (50.0) | 11/16 (68.8) | 0.440 | 0.292 | (-0.190 to 1.070) | 0.156 | **-0.095** | **0.041** | **(-0.183 to -0.007)** | **0.037** |
| Gentamicin (%) | 5/18 (27.8) | 3/16 (18.8) | -0.328 | 0.294 | (-0.963 to 0.307) | 0.285 | **-0.094** | **0.040** | **(-0.180 to -0.008)** | **0.034** |
| ESBL production (%) | 7/18 (38.9) | 11/16 (68.8) | -0.002 | 0.303 | (-0.656 to 0.652) | 0.995 | -0.002 | 0.049 | (-0.107 to 0.103) | 0.970 |
| Imipenem (%) | 0/18 (0.0) | 0/16 (0.0) | - | - | - | - | - | - | - | - |
| ***Klebsiella pneumoniae*** |  |  |  |  |  |  |  |  |  |  |
| Ciprofloxacin (%) | 12/16 (75.0) | 3/7 (42.9) | **1.385** | **0.340** | **(0.304 to 2.465)** | **0.027** | **-0.351** | **0.035** | **(-0.462 to -0.241)** | **0.002** |
| Gentamicin (%) | 0/16 (0.0) | 0/7 (0.0) | - | - | **-** | - | - | - | **-** | - |
| ESBL production (%) | 12/16 (75.0) | 4/7 (57.1) | **2.135** | **0.340** | **(1.054 to 3.215)** | **0.008** | **-0.434** | **0.035** | **(-0.545 to -0.324)** | **0.001** |
| Imipenem (%) | 0/16 (0.0) | 0/7 (0.0) | - | - | **-** | - | - | - | **-** | - |
| ***Acinetobacter baumanii*** |  |  |  |  |  |  |  |  |  |  |
| Ciprofloxacin (%) | 10/12 (83.3) | 3/4 (75.0) | **0.501** | **0.160** | **(0.123 to 0.878)** | **0.016** | **-0.131** | **0.020** | **(-0.178 to -0.084)** | **0.000** |
| Gentamicin (%) | 10/12 (83.3) | 3/4 (75.0) | **0.501** | **0.160** | **(0.123 to 0.878)** | **0.016** | **-0.131** | **0.020** | **(-0.178 to -0.084)** | **0.000** |
| Cefepime (%) | 10/12 (83.3) | 3/4 (75.0) | **0.501** | **0.160** | **(0.123 to 0.878)** | **0.016** | **-0.131** | **0.020** | **(-0.178 to -0.084)** | **0.000** |
| Imipenem (%) | 9/12 (75.0) | 3/4 (75.0) | **0.727** | **0.252** | **(0.130 to 1.323)** | **0.024** | **-0.102** | **0.032** | **(-0.178 to -0.027)** | **0.015** |
| ***Pseudomonas aeruginosa*** |  |  |  |  |  |  |  |  |  |  |
| Ciprofloxacin (%) | 12/12 (100) | 15/18 (83.3) | **-1.184** | **0.298** | (-1.847 to -0.520) | **0.003** | 0.059 | 0.045 | (-0.043 to 0.160) | 0.227 |
| Gentamicin (%) | 12/12 (100) | 14/18 (77.8) | **-1.256** | **0.269** | (-1.856 to -0.657) | **0.001** | 0.043 | 0.037 | (-0.040 to 0.125) | 0.276 |
| Cefepime (%) | 12/12 (100) | 18/18 (100) | - | - | - | - | - | - | - | - |
| Imipenem (%)v | 12/12 (100) | 12/18 (66.7) | **-0.785** | **0.332** | **(-1.525 to -0.045)** | **0.040** | 0.040 | 0.039 | (-0.048 to 0.127) | 0.335 |
| ***Staphylococcus aureus*** |  |  |  |  |  |  |  |  |  |  |
| Ciprofloxacin (%) | 6/10 (60.0) | 3/4 (75.0) | -0.441 | 0.316 | (-1.799 to 0.917) | 0.297 | -0.190 | 0.081 | (-0.538 to 0.158) | 0.144 |
| Gentamicin (%) | 6/10 (60.0) | 2/4 (50.0) | -0.766 | 0.574 | (-3.234 to 1.702) | 0.314 | -0.150 | 0.097 | (-0.569 to -0.268) | 0.262 |
| Oxacillin (%) | 6/10 (60.0) | 3/4 (75.0) | -0.441 | 0.316 | (-1.799 to 0.917) | 0.297 | -0.190 | 0.081 | (-0.538 to 0.158) | 0.144 |
| ***Enterococcus faecium*** |  |  |  |  |  |  |  |  |  |  |
| Ampicillin (%) | 3/3 (100) | 3/3 (100) | -0.885 | 0.680 | (-3.810 to 2.041) | 0.323 | -0.192 | 0.141 | (-0.800 to 0.416) | 0.307 |
| Ciprofloxacin (%) | 3/3 (100) | 3/3 (100) | -0.885 | 0.680 | (-3.810 to 2.041) | 0.323 | -0.192 | 0.141 | (-0.800 to 0.416) | 0.307 |
| Vancomycin (%) | 2/3 (66.7) | 3/3 (100) | -1.984 | 0.680 | (-4.910 to 0.943) | 0.100 | -0.088 | 0.141 | (-0.697 to 0.521) | 0.598 |

C) Sputum isolates

|  | Pre-intervention period | Intervention period | Change in level | SE | 95% CI | *P* | Change  in trend | SE | 95% CI | *P* |
| --- | --- | --- | --- | --- | --- | --- | --- | --- | --- | --- |
| ***Escherichia coli*** |  |  |  |  |  |  |  |  |  |  |
| Ciprofloxacin (%) | 6/16 (37.5) | 40/42 (95.2) | 0.398 | 0.276 | (-0.195 to 0.991) | 0.171 | **-0.111** | **0.040** | **(-0.196 to -0.025)** | **0.015** |
| Gentamicin (%) | 4/16 (25.0) | 37/42 (88.1) | -0.283 | 0.316 | (-0.960 to 0.394) | 0.385 | -0.051 | 0.043 | (-0.145 to 0.042) | 0.255 |
| ESBL production (%) | 9/16 (56.3) | 32/41 (78.0) | 0.122 | 0.349 | (-0.627 to 0.871) | 0.732 | 0.012 | 0.051 | (-0.097 to 0.121) | 0.813 |
| Imipenem (%) | 0/16 (0.0) | 0/42 (0.0) | - | - | - | - | - | - | - | - |
| ***Klebsiella pneumoniae*** |  |  |  |  |  |  |  |  |  |  |
| Ciprofloxacin (%) | 16/33 (48.5) | 45/63 (71.4) | 1.648 | 0.554 | (-0.116 to 3.413) | 0.059 | **-0.273** | **0.072** | **(-0.503 to -0.042)** | **0.033** |
| Gentamicin (%) | 2/33 (6.1) | 0/63 (0.0) | - | - | - | - | - | - | - | - |
| ESBL production (%) | 15/33 (45.5) | 47/63 (74.6) | **2.398** | **0.554** | **(0.634 to 4.163)** | **0.023** | **-0.356** | **0.072** | **(-0.586 to -0.126)** | **0.016** |
| Imipenem (%) | 1/33 (3.0) | 0/63 (0.0) | - | - | - | - | - | - | - | - |
| ***Acinetobacter baumanii*** |  |  |  |  |  |  |  |  |  |  |
| Ciprofloxacin (%) | 246/246 (100) | 148/150 (98.7) | -0.009 | 0.286 | (-0.919 to 0.901) | 0.977 | - | - | - | - |
| Gentamicin (%) | 237/246 (96.3) | 120/150 (80.0) | -0.009 | 0.286 | (-0.919 to 0.901) | 0.977 | - | - | - | - |
| Cefepime (%) | 244/246 (99.2) | 148/150 (98.7) | -0.009 | 0.286 | (-0.919 to 0.901) | 0.977 | - | - | - | - |
| Imipenem (%) | 244/246 (99.2) | 146/150 (97.3) | **0.754** | **0.234** | **(0.009 to 1.500)** | **0.049** | - | - | - | - |
| ***Pseudomonas aeruginosa*** |  |  |  |  |  |  |  |  |  |  |
| Ciprofloxacin (%) | 36/60 (60.0) | 81/118 (68.6) | **-0.866** | **0.358** | **(-1.664 to -0.068)** | **0.036** | 0.016 | 0.045 | (-0.084 to 0.116) | 0.723 |
| Gentamicin (%) | 33/60 (55.0) | 64/118 (54.2) | **-0.962** | **0.329** | **(-1.695 to -0.228)** | **0.015** | 0.005 | 0.037 | (-0.079 to 0.088) | 0.906 |
| Cefepime (%) | 60/60 (100) | 118/118 (100) | - | - | - | - | - | - | - | - |
| Imipenem (%)v | 38/60 (63.3) | 71/118 (60.2) | -0.498 | 0.374 | (-1.331 to 0.336) | 0.213 | 0.003 | 0.041 | (-0.088 to 0.095) | 0.938 |
| ***Staphylococcus aureus*** |  |  |  |  |  |  |  |  |  |  |
| Ciprofloxacin (%) | 93/106 (87.7) | 41/66 (62.1) | -0.649 | 0.613 | (-3.288 to 1.991) | 0.401 | 0.454 | 0.126 | (-0.089 to 0.997) | 0.069 |
| Gentamicin (%) | 56/106 (52.8) | 28/66 (42.4) | -1.649 | 0.613 | (-4.288 to 0.991) | 0.115 | **0.954** | **0.126** | **(0.411 to 1.497)** | **0.017** |
| Oxacillin (%) | 92/106 (86.8) | 49/66 (74.2) | -0.649 | 0.613 | (-3.288 to 1.991) | 0.401 | 0.454 | 0.126 | (-0.089 to 0.997) | 0.069 |
| ***Enterococcus faecium*** |  |  |  |  |  |  |  |  |  |  |
| Ampicillin (%) | 15/15 (100) | 2/2 (100) | - | - | - | - | - | - | - | - |
| Ciprofloxacin (%) | 15/15 (100) | 2/2 (100) | - | - | - | - | - | - | - | - |
| Vancomycin (%) | 9/15 (60.0) | 1/2 (50.0) | -1.932 | 0.532 | (-4.220 to 0.355) | 0.068 | -0.062 | 0.068 | (-0.356 to 0.232) | 0.459 |

^a^ The unit for change in level is antimicrobial resistance rate (%); the unit for change in trend is antimicrobial resistance rate (%) per month

^b^ Abbreviations: SE, Standard errors; CI, Confidence interval; ESBL, Extended-spectrum beta-lactamase
